# Exploring Coping Strategies of Different Generations of Students Starting University

**DOI:** 10.3389/fpsyg.2021.740569

**Published:** 2021-09-30

**Authors:** Rita Takács, Szabolcs Takács, Judit T Kárász, Zoltán Horváth, Attila Oláh

**Affiliations:** ^1^Institute of Psychology, Eötvös Loránd University, Budapest, Hungary; ^2^Doctoral School of Psychology, Eötvös Loránd University, Budapest, Hungary; ^3^Institute of Psychology, Department of General Psychology and Methodology, Károli Gáspár University of the Reformed Church, Budapest, Hungary; ^4^Doctoral School of Education, Eötvös Loránd University, Budapest, Hungary; ^5^Institute of Education, Eötvös Loránd University, Budapest, Hungary; ^6^Institute of Computer Science, Eötvös Loránd University, Budapest, Hungary

**Keywords:** university student, COVID-19, Generation Z, Generation Y, psychological immune system inventory questionnaire

## Abstract

**Introduction:** Coping strategies and adaptation skills are key features in successfully adjusting to university challenges. Coping skills are an essential part of the Psychological immune system, which leads to successful adaptation. Due to COVID-19 most universities have changed their face-to-face teaching for online education. Nevertheless, there is little concrete empirical evidence on how this generation of students with the ongoing impacts of disruptive changes can cope with it. Colleges and universities need to make changes in order to retain this new generation of students. Our aim was to explore the characteristics and changes in coping skills of university students from three different age groups.

**Method:** Psychological coping skills were measured by the Psychological Immune Competence Inventory (PICI). Differences were detected between generations. Group comparisons (pre-2004, pre-Covid, and post-Covid) groups were compared) using PICI subscales using independent sample analysis of variance. The sample consisted of 4,731 university students, 2,768 (58.5%) were men and 1,730 (36.56%) were women.

**Results:** Students from 2004 showed significantly higher scores in the Self-regulation subsystem scale compared to students in the pre-Covid and post-Covid groups. Self-regulation subsystem: *F*(2, 2,569.607) = 444.375, *p* < 0.001, η^2^ = 0.27: small effect, ω^2^ = 0.27; Resilience: *F*(2, 2,372.117) = 1171.855, *p* < 0.001, η^2^ = 0.14: small effect, ω^2^ = 0.14. Based on the results, the explained variance ratio was at least 10% based on self-regulation and resilience.

**Conclusions:** Psychological immune capacity of students seems to decrease through the years. Nonetheless, interventions may have a further facilitating role in the maintenance and development of psychological immunity during college years.

## Introduction

### Literature Review

The meta-analyses conducted strongly predict the success of higher education students based on past school performance, socioeconomic status (e.g., Sackett et al., 2009), parental education, higher intelligence, and learning and self-regulatory strategies (e.g., Richardson et al., 2012). Schneider and Preckel (2017) conducted a review of variables associated with achievement in higher education in their study, thus providing a systematic and comprehensive overview of the international higher education research literature. The variables of all empirical studies related to student performance over the past two decades have been examined. The study described 105 variables that contained data from nearly two million students. The review allowed different educational methods and institutions of higher education to be compared based on impact indicators. According to that study, higher education research has two central questions: one about teaching methods and the other about attributes of students that can predict their academic performance. The following characteristics describe students who perform well during their studies in higher education: self-efficacy, intelligence, and targeted use of learning strategies.

Researchers have been analyzing and discussing about how students cope with stress in their academic lives for a very long time and it is essential to continue to understand this issue. Many researchers have also studied the relationship between psychological characteristics and dropout from higher education. According to Chen (2008) there is a relationship between coping strategies and psychological well-being in college. It has been proved that positive coping strategies had significant buffering effects on psychological health problems. There are various studies which showed that in general, self-efficacy is one of the main coping strategies that helps to improve university students’ performance (Freire et al., 2020).

### Coping With University Stress

Coping with challenges during academic studies is affected by a large number of factors. Dealing with stress, anxiety, and/or difficult emotions at university can interfere with the ability to pay attention, learn new information. Stress factors can be: lack of time, overloaded curriculum, tests, perfectionism (i.e., setting expectations toward oneself too high), competitiveness among students and family problems.

Towbes and Cohen (1996) claim that stress could be a major issue for university students as they have to adapt to academical, social and individual challenges. Most students face continuous pressure for a good academic performance (Oman et al., 2008). Although the concept of coping strategies is still a controversial issue (Stanisławski, 2019), numerous distinctive adapting behaviors have been considered within the long history of research about stress. Some of the coping behaviors are considered more “active,” i.e., cognitive reframing (Tobin et al., 1989), whereas other strategies are more “passive” (e.g., social withdrawal). Some other coping behaviors have been identified during collective crises by Fullana et al. (2020); for example, following routines or maintaining healthy habits during COVID-19 breakdown.

Lazarus claims that according to the basic concept of coping, we are in a constant, two-way interaction with our environment (Lazarus, 1993). Coping is an assessment process designed to respond to external and internal challenges. Coping strategies are defined as efforts to regulate emotions, behaviors, cognitions and environmental aspects in response to the stress of everyday events. Each situation requires the use of a specific coping strategy. In many cases, managing the situations exceeds the resources the individual has. When facing a difficult situation, we evaluate how threatening or challenging the situation is according to our own goals and resources. And then we activate our resources to handle it and apply coping strategies. We distinguish several coping strategies. Problem-focused coping is activated when the situation is evaluated as changeable and controllable and we strive to focus on the problem solution. Emotion-focused strategies are activated when the situation appears unchangeable and we seek to reduce our negative feelings. There are even other classifications of coping strategies, such as social support. It is important to map and develop our coping strategies in order to activate the most effective coping strategies during difficult situations to deal with stressors. In dealing with crises that unfold in everyday life situations, we mobilize our psychological competencies. As Galiana et al. (2020) claims, even by elder people coping strategies have a great impact on well-being.

Higher education students may confront numerous unpleasant challenges in higher education. Denovan and Macaskill (2013) claim that college students apply many types of coping mechanisms e.g., self-control, trust, and positive thinking in order to better adjust to stressful situations. The type of coping strategy used depends on the perceived level of self-efficacy by the individual (Vandercleyen et al., 2014; Zambianchi and Ricci-Bitti, 2014; Gárriz et al., 2015).

Being aware of our strength helps to cope mentally and emotionally with stress at university. Coping strategies can be: respecting your limits, setting priorities, avoiding comparisons, leisure activities (watching movies, literature, sport, meeting with friends), assertiveness, building community, cognitive restructuring, and social networking. Li et al. (2018) investigated 262 university students in China and established that self-esteem had a mediating role in the relationship between social support and academic achievement. The main objective of the study of Morales-Rodríguez and Pérez-Mármol (2019) was to explore if anxiety, coping strategies, and emotional intelligence were related to the levels of self-efficacy in a sample of Spanish university students (*N* = 258). According to the results, general perceived self-efficacy is statistically related to state and trait anxiety, and to coping strategies of problemsolving, emotional expression, cognitive restructuring and social withdrawal. Kotera et al. (2021) got similar results, they compared Malaysian (*N* = 153) and United Kingdom students (*N* = 105) by paper-based measures about mental health problems, negative mental health attitudes, self-compassion, and resilience. Mental health problems were positively associated with negative mental health attitudes, and negatively associated with self-compassion and resilience. As a result of the survey, self-compassion training was suggested to university students for improving their mental health.

The results show that putting effort by the institution into developing core skills can have a developing effect on self-esteem which according to the results fully mediates the relationship between social support and academic achievement (Bredács, 2016).

### Psychological Immune System

In dealing with crises in daily life, the individual mobilizes his or her psychological competencies. The adaptive and maladaptive coping are activated along self-efficacy: the higher the self-efficacy skills, the stronger coping strategies will be activated. Psychological immune competence, as defined by Oláh (2009), is a collective concept of psychological characteristics based on the defense system of the personality. The psychological immune system protects the personality from the damaging effects of stress on physical and mental health (Oláh, 2005; Oláh et al., 2010; Bredács, 2016). At what level and with what results a person is able to cope with stress depends largely on his or her psychological immune competence, the inviolability and unpredictability of the situation, and the coping capacity of the personality (Jaiswal et al., 2020). Coping is one of the main variables that moderates the dynamic interaction between stress from the environment and the response elicited by the individual (Oláh, 2005). Two forms of coping can be distinguished: problem-focused and emotion-focused. Problem-centered coping means that the individual tries to achieve change by focusing on the problem, either in the environment or in themselves. In the case of emotion-focused coping, the individual is not able to deal directly with the problem, but by using different strategies (conscious or unconscious) tries to eliminate the psychological pressure evoked by the situation (Oláh, 2009; Kaur and Som, 2020). According to Oláh (2005), cognitive personality traits contribute to an individual’s successful coping. In his concept, he created a broad and comprehensive system, a set of personality traits that help with coping, which he called the psychological immune system. The psychological immune system unites personality resources that help the individual to endure and cope with a stressful situation, while the person’s integrity and developmental potential are not damaged, but strengthened during the active stress response of the situation. The psychological immune system provides active protection against external and internal factors that may hinder the individual’s integrated functioning. The psychological immune system is composed of three subsystems: approach-belief subsystem, monitoring-creating-executing, and self-regulatory subsystem. Units of the approach-belief subsystem are optimism, a sense of coherence, the ability to seek challenges, and the ability to monitor the physical and social environment by tuning the cognitive apparatus to positive consequences. The components of the monitoring-creating-executing subsystem are ingenuity, problem-solving ability, self-efficacy, and the ability to mobilize social resources and social creativity. The subsystem unites the personality traits by mobilizing, and thus enables an individual to shape their environment or themselves according to their goals. The self-regulatory subsystem includes coping potentials that provide control over attention and conscious functioning, which are: synchronicity (directing attention and keeping the focus to reach the desired goal), persistence, irritability inhibition, impulsivity control, and emotional control. The three subsystems interact dynamically with each other, thus stimulating and regulating each other’s functioning and enabling the self’s development and fulfillment through the integration of self-seeking information (Oláh, 2005). The development of a well functioning psychological immune competence in education should be a key factor to student development (Bredács, 2016). Oláh (2009) draws attention to higher education that those who are able to set meaningful goals for themselves and see the meaning of the work invested are able to trust in the efficiency and development of their own skills, evaluate themselves more positively and consider themselves persistent enough to achieve. A low psychological immune competence can be an indicator of anxiety and lack of self-confidence. Oláh et al. (2010) also studied participants from different cultures and confirmed that those who manage to set clear goals for themselves are better aware of their own abilities, and thus can effectively mobilize the energy resources needed to perform the tasks. The more they can be in harmony with their own abilities and feel to have a sense of control, the less they will feel unstable or anxious, and their perseverance and satisfaction will increase. As part of the process, their own talent and self-efficacy can unfold.

The conception of psychological immune system has various related studies to factors in the field of health psychology. Recent research surveying psychological immune system among emergency nurses (Gombor, 2009), gymnasts (Bóna, 2014), and military soldiers (Hullám, 2005), showed that psychological immune competence had a positive correlation with life satisfaction and well-being measurements (e.g., personal growth, self-acceptance, purpose in life) and a negative correlation with burnout (Oláh, 2009). Voitkâne (2004) showed that a significant relationship existed between psychological immune subsystems and personal goals. Gombor (2009) involved Swedish nurses in his study and the results showed that psychological immune system was the best protective factor against burnout. Furthermore, psychological immune competence is strongly correlated with mental and physical health (Oláh, 2009), with the hope of attaining goals, with life satisfaction, life expectancy (Oláh et al., 2010) and negatively with depression (Voitkâne, 2004).

### Millennials and Gen Z

According to Mannheim (1952), a generation is a group of people of the same age in a similar social location experiencing similar social events (in Pilcher, 1994). Articles and books focus on the characteristics of generations in colleges and universities with the belief that generations differ in values, attitudes toward studying and behaviors (Gabrielova and Buchko, 2021). Higher education is full of challenges and it is essential to analyze coping mechanisms to understand generations in order to retain them at the university. We focus in this article on the two youngest generations, Millennials and Generation Z.

The generation which was born 1995–2012 is called Generation Z (McCrindle and Wolfinger, 2014), and is an interesting crossover from the previous Generation Y (or Millennials). The birth period of the previous generation is 1981–1995 (National Endowment for Financial Education, 2015). They are called Millennials because they were raised in the digital age, a sign of the upcoming new millennium (Anderson and Jiang, 2018). We can also call Generation Z as iGeneration because they always had access to the internet, iPods, and iPhones. This immediate ability to retrieve and transmit information could have a strong influence on their thinking and learning methods. Given the size of this group of people, it is perhaps not surprising that much effort has been devoted to understand them and seeking to improve their skills as students.

The first of the Generation Z cohort started graduating from high school in 2013, and college in 2017 or are still studying.

While Generation Z shares many traits with the millennial generation, they also bring in new patterns of behaviors at the university (Iorgulescu, 2016). Like Millennials, they are interested in obtaining new information quickly. Many Millennials need to be trained to develop essential studying skills, because Generation Z has been less involved in face-to-face communication. They want to be socially connected with everyone (Turner, 2015).

Generation Z is a kind of generation growing up with a culture of overprotective parents, a generation that has not received the opportunity to develop proper life management skills (Lukianoff and Haidt, 2015). Becoming a self-conscious individual involves making decisions and taking responsibilities for actions in uncertain situations and under unknown circumstances. Having overprotective parents hindered them in their proper social, emotional and intellectual development, which serves as an obstacle to be able to explore challenges of life and navigate in different working environments such as universities and colleges (Turner, 2015; Gabrielova and Buchko, 2021). There is little information about how Generation Z is going to be influenced by COVID-19.

### Covid-19 and Its Effect on This Generation

Currently, there is little literature about COVID-19 in relation to how it effects coping skills at universities, so it is worth discussing it and have an overview of recent studies about students’ mental health.

Some researchers started to examine the effects of COVID-19 pandemic on university students’ mental health. Browning et al. (2021) highlight that university students are increasingly considered a vulnerable population, since they experience extremely high levels of anxiety and depression. As the education has changed radically due to COVID-19 pandemic, it calls attention to the fact that students suffer with more mental health problems. Padrón et al. (2021) applied a path-analysis model integrating stressors, coping, and mental health. According to their results, coping strategies partially were a mediator valuable between the effect of stressors and psychological health. Agbaria and Mokh (2021) investigated the relationships between active, problem-focused, and maladaptive coping with stress during the first wave of coronavirus outbreak among college students. They found that positive social support may increase students’ ability to cope effectively with the current situation. Another interesting research was conducted by Vitales et al. (2021). One-hundred males and one-hundred females from each generation participated in the survey (Baby Boomers, Generation Y, Generation X, and Generation Z). It was only their psychological-spiritual coping strategies where they found significant difference among the generations. Arora et al. (2021) examined the impact of coronavirus and online education on students’ anxiety and self-efficacy, and they found that coping strategies had a moderating role between anxiety and self-efficacy. The correlation was lower in students with higher levels of coping strategies (self-efficacy) than in students with lower levels of coping strategies (self-efficacy). Nomura et al. (2021) also reported that interventions should be made because COVID-19 had an effect on the prevalence of depressive symptoms as well as suicide-related ideation among Japanese university students. Szczepańska and Pietrzyka (2021) found a strong correlation (*N* = 135) between the severity of lockdown measures during COVID-19 pandemic and the students’ activity levels in public spaces. Students were affected by the absence of direct social interactions, which caused a considerable deterioration in students’ physical and psychological well-being, and the overall quality of life (Szczepańska and Pietrzyka, 2021). Coping with stress among graduate and professional students (*N* = 305) during the lockdown was also discussed by Wasil et al. (2021). Students reported top problems relating to productivity (27% of sample), physical health (26%), and emotional health (14%). As a coping strategy movement activities (like sport) were the most frequently identified (50%). Students who reported a common strategy had lower depressive and anxiety symptoms. In general, results suggest that students’ psychological health was substantially affected by the COVID-19 pandemic situation and that the academic and relational changes were the most notable sources of stress.

Gonzalez et al. (2020) analyzed the effects of COVID-19 on the learning performance of students. The results show that there has been a significant positive effect of the COVID-19 restrictions on students’ academic performance: students have improved their strategies of learning. They expected better scores in students’ effectiveness. Lee et al. (2021) analyzed a student course satisfaction survey, conducted during the 2020 summer term, and it appeared that due to COVID-19 students were more resilient during the first lockdown than was often assumed. These studies reinforce the need to monitor and promote mental health in university students to boost their resilience in times of crisis.

Based on the literature above, there are two major immediate needs for high quality research to be conducted. Nevertheless, research on coping skills of Generation Z are still limited, and in order to better adjust the higher education to students’ needs not just during the COVID-19 crisis but also in general, the impact of the various types of coping skills on students’ adjustment is largely unknown (Apgar and Cadmus, 2021).

Research on stress among students and its effects have been well-documented from various perspectives. Researchers agree that students share common academic stressors such as time-management, exam anxiety and course related stress (Malarvili and Dhanapal, 2018). However, there is a lack of studies comparing the perceptions of Generations Y and Z regarding coping skills, especially in the field of higher education. Thus, this quantitative study aimed to identify the difference between coping skills of Generations Y and Z students. These are key factors of coping with university stress and maintaining mental health. For the purpose of this study, Generation Y are those born between 1981 and 1994, while Generation Z includes those born from 1995 to 2012 (McCrindle and Wolfinger, 2014). The reason for choosing Generations Y and Z as the sample is due to the fact that these are the latest generations who entered higher education and the difference among them can lead us to recommendations on what we should change in higher education in order to improve their coping skills. Understanding the differences and their strengths might help us to find new methods that support the current generation to successfully adapt to academic and life challenges.

This article examines the coping skills of today’s generation of students in order to provide new perspectives on how different staff members of the university can support Generation Z in their academic success. The result of this discussion is significant because the results could be a remedy for the concerns of administrators, faculty members, teachers and practitioners on how to apply intervention strategies.

The main aim of the study was to examine the differences between Generation Y- from 1981 to 1994- and Z- from 1995 to 2012- regarding to the self-regulation and resilience subsystem. Self-regulation subsystem is related to emotional control, perseverance, impulse and irritability control, thus this system helps dealing with stress-related tensions. Being less resilient can lead to insufficiency in adapting to changes in the environment which means that the younger generation could have problems with adaptation and difficulties to deal with academic and interpersonal challenges. It is self-regulation that allows coping with stress and controlling feelings. The self-regulation subsystem is in control of the goals achievement and controlling feelings after failure. This subsystem can interfere within the young people that present more difficulty moving away from bad feelings related to discomfort. This population can be identified as risky because they are less capable of mobilizing social resources or efficient tools of stress management.

### Research Hypotheses

The research hypotheses of the current study are:

H1: Generation Z has significantly stronger approach-belief subsystem than the Generation Y.

H2: Generation Z has significantly stronger creative-executive social and individual effectiveness (Monitoring- Creating- Executing subsystem) than Generation Y (2004s).

H3: Generation Y has significantly stronger self-regulation than Generation Z.

H4: Generation Y has significantly stronger resilience than Generation Z.

H5: There is no significant difference between pre- and post-COVID generations’ psychological immune systems.

## Materials and Methods

### Participants

The global sample consisted initially of 4,731 first-year university students recruited from various academic areas. In the final sample, 2,768 (58.5%) were men and 1,730 (36.56%) were women, 233 participants did not indicate their biological sex (4.9%). The mean age of the participants was 20.06 years; and the age range was between 16 and 51 years. The inclusion criterion was to be first-year student at the time of the study. Exclusion criteria included failing to respond to the questionnaire. We excluded 233 cases because they failed to respond to enough items. The students in the sample studied in the academic areas of humanities (33%), computer science and engineering (64%).

### Procedures

The study protocol was designed and executed in compliance with the code of ethics set out by the university in which the research was conducted, with the informed consent of all participants, as required by the Helsinki Declaration. Participants were assured of anonymity and the confidentiality of their responses.

In 2004 undergraduate first-year students were approached at the beginning of the semester. They were asked to complete a hard copy of the questionnaire. The participants were informed that the data collected would remain anonymous and used only for research purposes. It took the participants an average 20 min to complete the self-report questionnaire.

Participants from Generation Z (in each October beginning from the semester 2013 until 2020) filled out the questionnaires using an internal web application. Students were invited to participate in filling in the questionnaire mainly at an academic course (Preparation course for academic studies and learning strategies) and the students were also encouraged to spread the link to fellow students using the same platform.

The present study protocol was approved by the Ethics Committee of the University of Anonymous, with the registration number: 61, and prior to beginning the questionnaire, participants were provided with the goal and requirements of the study. They were also asked to give their explicit agreement to participate in the study and were informed that participation was completely anonymous and voluntary. On average, the survey took 20 min to complete and there was no reward or compensation for participating. The first year of university education is the hardest because students have to face unexpected and unknown difficulties.

### Data Analyses

Group comparisons (pre-2004, pre-Covid, and post-Covid) groups were compared) using PICI subscales using independent sample analysis of variance. The homogeneity of variances was tested with Levene’s test, and in the case of damage to the homogeneity of variances, the Welch’s analysis of variance was used. In addition to significance, effect variance (eta-square and omega-square) indicators were also calculated in each case. Kruskal–Wallis H test was used for testing whether there were differences between the examined groups.

### Instrument: Psychological Immune Competence Inventory Survey

Psychological immunity can be measured with the psychological immune competence questionnaire (Oláh, 2005). This questionnaire contains 16 scales for measuring personal protective characteristics. Participants were asked to respond to each item on a 4-point Likert scale (1 = “does not describe me at all” and 4 = “fully describes me”), indicating how well the statement describes them. The higher a person scores on the scale, the stronger is his/her psychological immune system. In this research, we used subscales of approach-belief subsystem, monitoring-creating-executing subsystem, self-regulating subsystem and resilience. One example of an item from the managed instrument: “When I look at my life, I see it evolving meaningfully and consistently.” Resilience is the ability that helps us cope with stress, and to reduce the negative effects of stress. A general accepted level of reliability is that α of 0.6–0.7, and 0.8 or greater is a very good level (Ursachi et al., 2015). The reliability of scales was at a very good level in this study (Table 1).

**TABLE 1:** Reliability measures of the psychological immune system inventory subscales.

Scale	Number of items	Cronbach alpha (α)	mean	variance
approach-belief subsystem	6	0.8	2.7	0.02
monitoring- creating- executing subsystem	4	0.76	2.91	0.02
self-regulating subsystem	3	0.79	2.6	0.1
resilience	3	0.77	2.63	0.09
psychological immune competence	16	0.88	2.74	0.05

Psychological Immune Competence Inventory showed high reliability and validity during testing. The descriptive statistics of the scales are in Table 2.

**TABLE 2:** Descriptive statistics of the scales.

Scales	Groups	*N*	Mean	Std. Deviation	Std. Error	Min	Max	95% Confidence Interval for Mean	
								Lower Bound	Upper Bound
Approach-belief subsystem	Generation Y (2004)	1,557	17.73	2.7	0.07	7	24	17.60	17.87
	Generation Z Pre-Covid (2013–2018)	1,557	15.93	4.44	0.11	0	24	15.70	16.15
	Generation Z Post-Covid (2019–2020)	1,232	15.68	5.01	0.14	0	24	15.40	15.96
	Total	4,346	16.51	4.2	0.06	0	24	16.38	16.63
Monitoring- creating- executing subsystem	Generation Y (2004)	1,565	12.63	1.66	0.04	5	16	12.55	12.71
	Generation Z Pre-Covid (2013–2018)	1,557	11.47	3.02	0.08	0	16	11.32	11.62
	Generation Z Post-Covid (2019–2020)	1,232	11.23	3.47	0.1	0	16	11.03	11.42
	Total	4,354	11.82	2.83	0.04	0	16	11.73	11.90
Self-regulating subsystem	Generation Y (2004)	1,560	9.86	1.76	0.05	3	12	9.77	9.94
	Generation Z Pre-Covid (2013–2018)	1,557	7.86	2.7	0.07	0	12	7.72	7.99
	Generation Z Post-Covid (2019–2020)	1,232	7.7	2.94	0.08	0	12	7.54	7.86
	Total	4,349	8.53	2.68	0.04	0	12	8.45	8.61
Resilience	Generation Y (2004)	1,559	10.87	1.31	0.03	3	12	10.81	10.94
	Generation Z Pre-Covid (2013–2018)	1,557	7.98	2.64	0.07	0	12	7.85	8.11
	Generation Z Post-Covid (2019–2020)	1,232	7.78	2.86	0.08	0	12	7.62	7.94
	Total	4,348	8.96	2.73	0.04	0	12	8.88	9.04
Psychological immune system	Generation Y (2004)	1,554	51.11	5.34	0.14	24	64	50.84	51.38
	Generation Z Pre-Covid (2013–2018)	1,557	43.23	10.35	0.26	0	63	42.72	43.75
	Generation Z Post-Covid (2019–2020)	1,232	42.39	12.27	0.35	0	64	41.70	43.07
	Total	4,343	45.81	10.35	0.16	0	64	45.50	46.12

The homogeneity of variances was impaired for all subscales according to the Levene test. Approach-belief subsystem [*F*(2, 4343) = 109.660, *p* < 0.001], monitoring-creating-executing subsystem [*F*(2, 4351) = 126.381, *p* < 0.001], self-regulating subsystem [*F*(2, 4346) = 158.797, *p* < 0.001], resilience [*F*(2, 4345) = 313.082, *p* < 0.001] at a significance level of 5%. Thus, Welch’s analysis of variance was used to compare the means of the groups.

## Results

Differences between groups were significant for all subscales based on the Welch test. Approach-belief subsystem: *F*(2, 2503,216) = 145.612, *p* < 0.001, η^2^ = 0.48: small effect; ω^2^ = 0.48. Monitoring- creating- executing subsystem: *F*(2, 2,419,480) = 146.151, *p* < 0.001, η^2^ = 0.47: small effect, ω^2^ = 0.47. Self-regulating subsystem: *F*(2, 2,569,607) = 444.375, *p* < 0.001, η^2^ = 0.27: small effect, ω^2^ = 0.27. Resilience: *F*(2, 2,372,117) = 1171.855, *p* < 0.001, η^2^ = 0.14: small effect, ω^2^ = 0.14.

These significant differences were also supported by the Kruskal–Wallis H test for all 4 subscales at *p* < 0.001. Result of the Kruskal–Wallis tests: approach-belief subsystem: *H* (2) = 186.856, *p* < 0.000; monitoring- creating- executing subsystem: *H* (2) = 169.996, *p* < 0.000; self-regulating subsystem: *H* (2) = 629.152, *p* < 0.000; resilience: *H* (2) = 1495.277, *p* < 0.000; psychological immune competence: *H* (2) = 892.047, *p* < 0.000.

We examined the differences between the generations based on the psychological immune systems:

Regarding the approach-belief subsystem, Generation Y had a higher average score than Generation Z, and the difference was significant; however, the explained variance ratio was at least 10%, which is why it should be discarded (Hypothesis 1). The analyzes performed were adequate to the proposed hypotheses and the differences between the compared groups were significant but the hypothesis was rejected because the explained variance was too small, and the effect sizes were small.

Considering the monitoring-creating-executing subsystem, Generation Y had a higher average score than Generation Z and the difference was significant; however, the explained variance ratio was at least 10%, which was why it was also to be discarded (Hypothesis 2). The analyzes performed were good enough to the proposed hypothesis and the differences between the compared groups were significant but the hypothesis was rejected due to the fact the explained variance was too small, and the effect sizes were small.

There were significant differences between the generations regarding the self-regulating subsystem (Hypothesis 3) and resilience (Hypothesis 4). Generation Y had significantly higher scores compared to the other younger generations.

We found no difference between the pre- and post-Covid generations regarding their psychological immune systems (Hypothesis 5). The confidence intervals can be found in Table 2 and there was no difference between the groups (pre- and post-Covid).

## Discussion

The monitoring-creating-executing subsystem is responsible for understanding and preparing actions to control the environment. Being open to positivity helps the enactment of positive coping strategies and discovering new solutions (Oláh, 2005, 2009). The monitoring-creating-executing subsystem can activate the access to the person’s resources and problem-solving capacity (Kaur and Som, 2020). Since the approach-belief subsystem consists of competences that are open to improvement, with appropriate interventions- like strengthening creativity, promoting to discover alternative solutions- students can be facilitated to change their attitudes, or map their possibilities whether they can make modifications in the environment (Bredács, 2016). The components which are part of the auto-regulating subsystem are the following: emotional control, perseverance, impulse control, irritability control, help in handling the tensions that arise due to stress, and also help coping by controlling feelings. The self-regulating subsystem is in control of the process of accomplishing goals, and helps to control emotional states after failure. The self-regulating subsystem score was lower for the younger generations, pre- and post- Covid generations (Generation Z). However, lower scores in the self-regulating subsystem allows us to see that they have difficulties in shutting out the discomfort evoked by negative feelings. They can be identified as a risk population as they are less capable of mobilizing social resources or effective stress management tools. The lower resilience can lead to insufficiency in adapting to the changing demands of the environment. It means that the younger generation (Z) could have adjustment problems, they can have difficulties to adapt to academic challenges. One of the conclusions could be that the younger generation needs prevention programs in order to strengthen their auto-regulating subsystem, their resilience and flexibility. There is a need to develop these competences of the psychological immune system that help the individual to experience more successful adaptation even in more demanding life situations like COVID-19.

When an individual is able to dive into completely whatever s/he is doing, s/he becomes involved in it (Bredács, 2016). This happens during academic activities and also while being with friends. Apathy, uneasiness, and other negative feelings might indicate adjustment problems and can appear as a potential risk for dropout at university.

There were no differences found in the other subsystems of the psychological immune system, namely the monitoring-creating-executing subsystem and the approach-belief subsystem, which suggests the following: Both generations (Y and Z) are able to find the entire world challenging and easily get involved in studying. The difference is in the self-regulation subsystem which refers to the fact that Generation Z is less able to control their feelings in stressful situations and have weaker self-regulation skills during academic activities. It is probable that generation Y has less capacity to transform negative feelings, evoked by life situations into constructive responses. These abilities can be strengthened by effective prevention programs. Our result is in concordance with the studies of younger generations (e.g., Oláh et al., 2010; Bredács, 2016) according to which the teenagers with stronger psychological immune systems are more likely to cope well with different situations.

Our research highlighted the strengths and weaknesses of the Generation Z at the university. The role of the psychological immune system in a healthy psychological profile and coping behavior is unarguable. Those who have stronger psychological immune competences can reframe challenges easier by giving them different, new meaning, and tackle difficult situations. The focus of this present study was a comparison of psychological immune system components in order to see the differences among students who entered the university between 2004 and 2020. Fundamental difference was found in the self-regulation subsystem and resilience. However, the new generation does not differ in the other factors analyised in this study (approach-belief subsystem and monitoring-creating-executing subsystem). It means that when facing new situations, students are able to see them as a challenge in a positive way and to cope with them creatively. The difference is that Generation Y is more impatient and they have a lack of ability to delay. They grew up in front of the computer and they are likely to get used to instant responses. They might become impatient if they do not find an information immediately, their tolerance level of not instant gratification is lower. Probably they have more difficulties in their interpersonal relationships. This kind of expectation of instant gratification when managing human relationships can easily result in frustration, because their expectations might differ from the reality. In this study differences between pre-Covid and post-Covid generations were not found, probably other psychological scales are needed to detect further differences.

A possible help could be in the education to promote mental health development at the university. Peer to peer programs also could be useful in order to strengthen coping mechanisms of students. There is a need for prevention programs that aim to develop the above mentioned abilities of university students, aiding better coping strategies with stress. Our study supports the idea of promoting prevention programs for all generations to strengthen their autoregulation skills and resilience.

### Limitations of the Study

The contributions of this study should be assessed, taking into account some limitations of the study. One of the limitation lies in the composition of the sample, which was dissimilar in terms of gender representation. The use of online self-reports as a data collection method may limit the validity of the results, since the questionnaires are applied on an online form which does not guarantee who is answering. The instrument applied is a self-report instrument which also could lead to some biases in responses.

## Conclusion

Various international studies mainly compare representatives of different generations, and less attention is paid to the psychological factors of adjustment at university within one generation. The aim of the present empirical study was to find differences in coping strategies among generations of students. As far as we know, there are limited studies focusing on analyzing different generations using the psychological immune system scales in the educational sector. However, knowing these differences would help to improve the learning process, to provide more efficient guidance, vocational-, counseling-, and other supporting services.

The present study was conducted to analyze the difference between psychological immune system subscales among university students in two generations (Z and Y). Our aim was to understand better how the psychological immune system can promote academic success.

The result reflects well the differences between the generations, how much they have changed over the past 20 years. This result shows that the young generation is fast in information processing but in the social dimension they are less effective compared to the other generation. These results could underline, in line with other researchers from the positive psychology context (e.g., Oláh, 2009), that flexibility in coping enhances university students’ perception of control over their challenges, making them feel better able to handle them. The results of our research may represent a significant contribution, in that they help increase our understanding the difference between generations.

When a difficult life situation occurs, the difference is also reflected in the various reactions of the generations. The pre-Covid and post-Covid generations did not differ regarding coping skills. The full capacity of the generations is still waiting to be discovered, but the data shows, when a life-threatening situation affects everyone, it will be reflected in the flexibility of adaptation.

The bigger issue is socialization, if Generation Z lacks their self-regulation skills, which appeared in the self-report questionnaire. It means that they are less flexible, or resilient as Generation Y used to be. Probably, COVID-19 is going to still weaken the ability of handling emotional experiences. The Generation Z might become emotionally unstable as a result of bad experiences. If an individual has difficulty managing their emotional tensions, the additional external stress makes it even more difficult. This can be interpreted that Generation Z is weaker in handling negative emotions compared to Generation Y.

The practical significance of the study lies in tailoring preventive educational programs to the results of this study. This study underpins the need to monitor and promote mental health of university students, especially to help to strengthen their resilience in times of a crisis, like the COVID-19 pandemic. Interventions could be designed that support self-regulation skills of students that helps to eliminate the negative effects of stress also in the educational setting, and thus can lead to better academic performances.

### Lines for Future Research

New studies are needed to expand the sample and use more rigorous study design (e.g., longitudinal studies). In order to make the results more generalizable to the university student population, future studies should use more thorough recruitment procedures that would give more balanced samples in terms of gender and field of studies. Participants should be recruited from different subject areas. In order to facilitate generalization of the results, new studies are needed which involve students from other countries and cultural contexts. Future studies could include not only questionnaires but also in-depth interviews with the students.

## Data Availability Statement

The raw data supporting the conclusions of this article will be made available by the authors, without undue reservation.

## Ethics Statement

The studies involving human participants were reviewed and approved by Eötvös Loránd University Psychological Ethical Committee. The patients/participants provided their written informed consent to participate in this study.

## Author Contributions

RT contributed to the conception and design of the study, led the data collection, analysis, and interpretation, and wrote and revised the manuscript. JT contributed to the design of the study, collected and interpreted the data. ST contributed to the design of the study, analyzed and interpreted the data, and revised the manuscript. ZH and AO contributed to the design of the study and revised the manuscript. All authors read and approved the final manuscript.

## Conflict of Interest

The authors declare that the research was conducted in the absence of any commercial or financial relationships that could be construed as a potential conflict of interest.

## Publisher’s Note

All claims expressed in this article are solely those of the authors and do not necessarily represent those of their affiliated organizations, or those of the publisher, the editors and the reviewers. Any product that may be evaluated in this article, or claim that may be made by its manufacturer, is not guaranteed or endorsed by the publisher.
